# Legume crop rotation suppressed nitrifying microbial community in a sugarcane cropping soil

**DOI:** 10.1038/s41598-017-17080-z

**Published:** 2017-12-01

**Authors:** Chanyarat Paungfoo-Lonhienne, Weijin Wang, Yun Kit Yeoh, Neil Halpin

**Affiliations:** 1 School of Agriculture and Food Sciences, The University of Queensland, St Lucia, QLD 4072 Australia; 2 Sustainable Organic Solutions Pty Ltd, Indooroopilly, QLD 4068 Australia; 3 Department of Science, Information Technology and Innovation, Brisbane, QLD 4001 Australia; 4 Environmental Futures Research Institute, Griffith University, Nathan, QLD 4111 Australia; 5 Australian Centre for Ecogenomics, School of Chemistry and Molecular Biosciences, The University of Queensland, St Lucia, QLD 4072 Australia; 6 Department of Agriculture and Fisheries, 49 Ashfield Rd, Bundaberg, QLD 4670 Australia

## Abstract

Nitrifying microorganisms play an important role in nitrogen (N) cycling in agricultural soils as nitrification leads to accumulation of nitrate (NO_3_^−^) that is readily lost through leaching and denitrification, particularly in high rainfall regions. Legume crop rotation in sugarcane farming systems can suppress soil pathogens and improve soil health, but its effects on soil nitrifying microorganisms are not well understood. Using shotgun metagenomic sequencing, we investigated the impact of two legume break crops, peanut (*Arachis hypogaea*) and soybean (*Glycine max*), on the nitrifying communities in a sugarcane cropping soil. Cropping with either legume substantially increased abundances of soil bacteria and archaea and altered the microbial community composition, but did not significantly alter species richness and evenness relative to a bare fallow treatment. The ammonia oxidisers were mostly archaeal rather than bacterial, and were 24–44% less abundant in the legume cropping soils compared to the bare fallow. Furthermore, abundances of the archaeal *amoA* gene encoding ammonia monooxygenase in the soybean and peanut cropping soils were only 30–35% of that in the bare fallow. These results warrant further investigation into the mechanisms driving responses of ammonia oxidising communities and their nitrification capacity in soil during legume cropping.

## Introduction

Sugarcane farms are mostly located in high rainfall (>1000 mm per year) tropics and subtropics. Fertiliser nitrogen (N) applied in such regions is susceptible to loss through processes such as denitrification and leaching, leading to nitrous oxide (N_2_O) emissions into the atmosphere and nitrate (NO_3_^−^) pollution in waterways, respectively^1,2^. In order to achieve high crop yields, the amount of N fertiliser applied to sugarcane crops are generally high (120 to 300 kg N ha^−1^ yr^−1^)^2,3^. In recent years, legume crop rotation during the fallow period between two consecutive sugarcane crop cycles has been promoted in Australia to improve soil health and to benefit from biological N_2_ fixation, thus reducing reliance on synthetic N for the subsequent crop^4,5^. Compared to the conventional practice of bare fallow or continuous cane as “plough-out replant”, legume rotation can improve soil fertility and suppress soil pathogens^6–8^. However, few studies have investigated the effects of legume rotation on soil microbiota and their function in relation to soil N cycling.

Nitrification is the microbe-mediated conversion of ammonium (NH_4_^+^) to nitrate (NO_3_^−^) which can be easily lost through leaching and denitrification, particularly in tropical or subtropical regions with high rainfall. One of the management strategies to enhance fertiliser N use efficiency and reduce its negative impact on the environment is to add nitrification inhibitors into NH_4_^+^-based fertilisers (including urea) or directly into soil^9,10^. Recent studies under controlled conditions found that certain plant species such as peanut, sorghum and grasses release phytochemicals from roots that inhibit activities of soil nitrifying microorganisms^11^. We hypothesised that compared to continuous mono-cropping or bare fallow, legume crop rotation may influence soil microbial community composition and the abundance of nitrifiers by altering soil N status and other bio-physico-chemical properties in the rhizosphere. Hence in the present study, we investigated possible impacts of two major rotational legume crops, peanut (*Arachis hypogaea*) and soybean (*Glycine max*), on soil nitrifying microbial communities in a sugarcane cropping soil under field conditions.

## Results and Discussion

### Soil Moisture and Mineral N Contents

Conventional soil tests demonstrated that soil moisture content did not differ significantly between the legume cropping and bare fallow soils at the time of sampling (Table 1). Initial soil NH_4_^+^ and NO_3_^−^ contents immediately before crop planting were 3.2 mg N kg^−1^ and 28.5 mg N kg^−1^, respectively. At the maximum biomass stage of the legume crops, NH_4_^+^ (1.2–4.0 mg N kg^−1^) was detected in the legume cropping treatments but not in the bare fallow. In contrast, NO_3_^−^ contents were significantly lower in the legume cropping treatments than in the bare fallow (Table 1). Accordingly, ratios of NH_4_^+^-N/NO_3_^−^N were consistently higher in the legume cropping soils compared to the bare fallow soil (*P* < 0.05). Indeed, soil mineral N content under field conditions could be affected by many factors such as N transformations, root uptake and N losses. The presence of NH_4_^+^ in the root zone of the legume crop plots and its corresponding absence in the bare fallow soil was likely due to slower NH_4_^+^ oxidation (nitrification) in the legume cropped soils and/or rhizodeposition of NH_4_^+^ from roots and nodules of the crops^12^.

**Table 1:** Selected soil physico-chemical properties under bare fallow and two legume rotational crops (peanut and soybean) at the time of soil sampling. Data represent averages ± SE of four replicates.

Soil properties	Bare fallow	Peanut cropping	Soybean cropping
Moisture (%)	9.0 ± 0.3^a^	6.5 ± 1.5^a^	8.2 ± 0.8^a^
pH (1:5, soil:H_2_O)	6.1 ± 0.2^a^	6.5 ± 0.2^a^	6.3 ± 0.0^a^
NH_4_^+^-N (mg kg^−1^)	0.0 ± 0.0^a^	1.2 ± 0.2^a^	4.0 ± 0.9^b^
NO_3_^−^-N (mg kg^−1^)	20.2 ± 3.9^a^	0.7 ± 0.3^b^	3.5 ± 0.7^b^
NH_4_^+^-N/NO_3_^−^-N ratio	0.0 ± 0.0^a^	1.7 ± 0.3^b^	1.2 ± 0.3^b^

Numbers within a row followed by different letters are significantly different (ANOVA, LSD post hoc test, at *P* < 0.05).

### Microbial Community Richness and Evenness

A total of 475,846,598 reads were sequenced from the twelve soil samples, of which 229,728 contained 16S rRNA gene sequences. Taxonomy was successfully inferred for 65,984 16S rRNA sequences, resulting in the identification of 1,261 OTUs (operational taxonomic units) . Based on these OTUs, there was no significant difference in the estimated microbial species richness (Chao1) between samples irrespective of treatment: 633 ± 18, 650 ± 19 and 499 ± 152 under bare fallow, peanut cropping, and soybean cropping, respectively. This is in agreement with findings in previous studies that legume crop rotation had little effects on soil microbial richness perhaps due to low diversity of the host-specific microbes associated with legumes relative to free-living microorganisms^13,14^. Similarly, Shannon’s index also indicated no significant difference in community evenness between treatments: 7.5 ± 0.1 (bare fallow), 7.7 ± 0.1 (peanut cropping) and 7.8 ± 0.0 (soybean cropping).

### Total Abundances of Bacteria and Archaea

Quantitative PCR results demonstrated significantly higher (*P* < 0.05) 16S rRNA gene copy numbers in the peanut (63.5 ± 13.3 × 10^8^ g^−1^ soil) and soybean (79.0 ± 4.0 × 10^8^ g^−1^ soil) treatments compared to the bare fallow (40.9 ± 4.80 × 10^8^ g^−1^ soil). Thus, the legume cropping increased the abundances of bacteria and archaea by 1.6 (peanut) and 2.0 (soybean) times compared to the bare fallow (*P* < 0.05). These increases in soil microbial biomass associated with legume cropping were consistent with findings in previous studies using other legumes such as black lentil, Tangier flatpea, chickling vetch and feed pea grown in cereal cropping rotation^6,15^, forage legumes in sugarcane rotation^16^, and in legume-grass intercropping^17,18^.

### Microbial Community Composition

**Figure 1:**
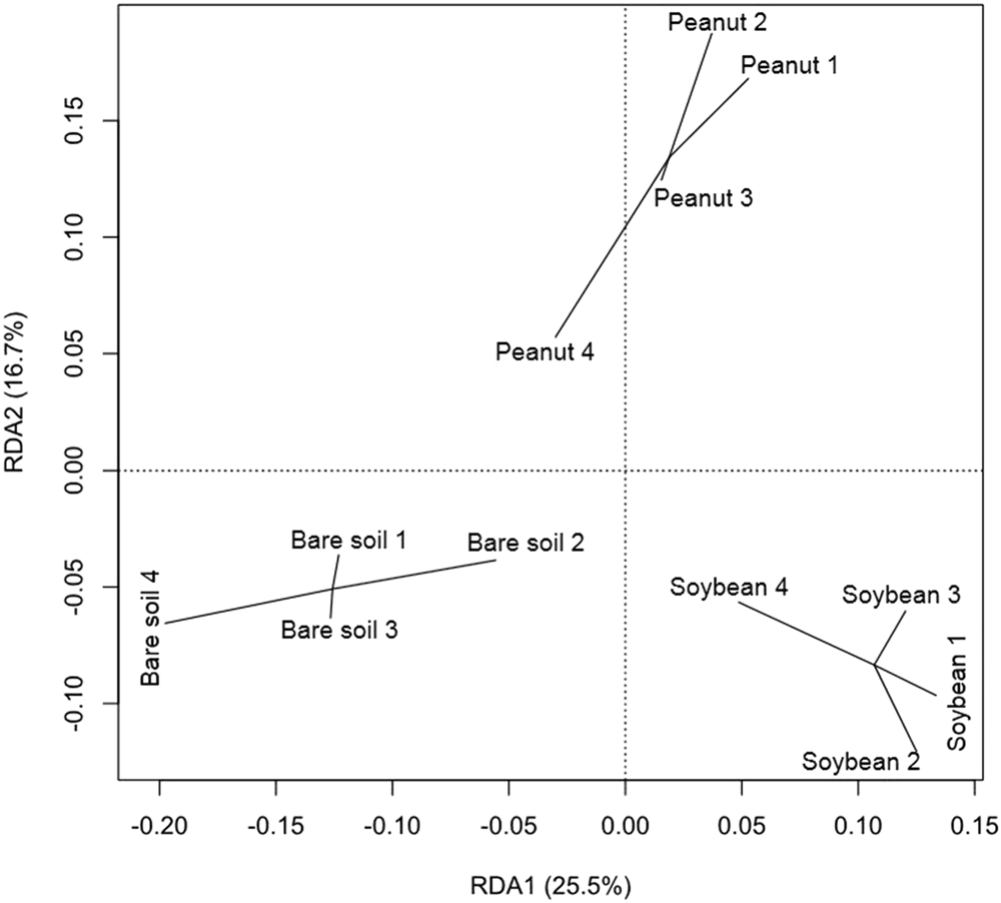
Ordination of soil microbial community composition in soils under different fallow management practices (Redundancy analysis, RDA). Replicates are connected to their respective group centroid. Components 1 and 2 represent 25.5% and 16.7% of the communities’ variance, respectively.

While the richness and evenness of microbial species were similar in different treatments, the overall microbial community composition significantly differed between the treatments (Fig. 1). This was in agreement with the previous work by Alvey *et al*.^16^, which also demonstrated that legume crop rotation has a substantial effect on the structure and diversity of soil microbial community. It was also noted that soil microbial community composition differed between soybean cropping and peanut cropping, suggesting that the crops imparted species-specific selective pressure on the surrounding soil microbial communities. This is not surprising since host species has been previously found to influence microbial community diversification in rhizosphere^19–21^. One of the proposed mechanisms for host-microbe interactions is rhizodeposition, where substrates from plant roots fuel microbial metabolism and subsequently drive community shift in the rhizosphere^20^.

In addition to crop species, other factors such as physico-chemical properties of soil, agricultural management, and microbe-microbe interactions can affect the soil microbial community^22^. Pearson’s correlation analyses showed that soil pH positively correlated to the abundances of bacteria and archaea (16S rRNA) (*r* = 0.64, *P < *0.05; *n* = 12) and microbial community evenness (Shannon’s index) (*r* = 0.69, *P* < 0.05; *n* = 12; Table 2). This result was consistent with previous findings that one of the most influential factors affecting the microbial abundance and community in soil is pH^23,24^.

**Table 2:** Pearson’s correlation coefficients (*n* = 12) between soil properties, soil microbial community and nitrification gene abundances.

	Moisture	pH	NH_4_^+^	NO_3_^−^	16S rRNA	Chao1	Shannon	AOA	AOB	NOB	*amoA* (AOA)	*amoA* (AOB)	*hao*	*nxrA*
pH	−0.59*	1.00												
NH_4_^+^	0.05	0.20	1.00											
NO_3_^−^	0.39	−0.75**	−0.45	1.00										
16S rRNA	−0.39	0.64*	0.48	−0.55	1.00									
Chao1	−0.34	0.00	−0.03	0.13	0.09	1.00								
Shannon	−0.46	0.69*	0.63*	−0.80**	0.65*	0.57	1.00							
AOA	0.06	−0.32	−0.75**	0.67*	−0.50	0.29	−0.59*	1.00						
AOB	0.13	0.11	0.67*	−0.08	0.06	0.15	0.46	−0.38	1.00					
NOB	0.34	−0.37	−0.43	0.14	−0.49	−0.30	−0.43	0.42	−0.60	1.00				
*amoA*(AOA)	0.45	−0.51	−0.66*	0.79**	−0.51	0.10	−0.60	0.77**	−0.16	0.28	1.00			
*amoA*(AOB)	0.11	−0.34	0.25	0.48	−0.03	0.17	−0.10	0.04	0.49	−0.30	0.14	1.00		
*hao*	−0.29	0.09	0.24	0.08	0.57	0.21	0.26	−0.29	0.21	−0.48	−0.05	0.41	1.00	
*nxrA*	−0.28	−0.34	−0.05	0.18	−0.34	0.19	−0.29	−0.16	0.03	−0.10	−0.24	0.38	0.15	1.00
*nxrB*	−0.48	0.11	0.15	−0.14	−0.01	0.05	0.10	−0.35	0.23	−0.28	−0.48	0.46	0.30	0.83**

*P < 0.05, **P < 0.01.

### Abundance of nitrifiers

The 16S rRNA gene sequence-based community composition indicated that the ammonia oxidisers responsible for conversion of ammonia to hydroxylamine in the first step of nitrification were mainly archaea rather than bacteria in this sugarcane cropping soil (Table 3). Relative abundances of these ammonia oxidisers were significantly lower (*P* < 0.05) in both the peanut (0.26 ± 0.08%) and soybean (0.20 ± 0.10%) cropped soils compared to the bare fallow (0.54 ± 0.15%). After taking into account the number of 16S rRNA genes measured in the soils, the absolute abundance of ammonia oxidisers in peanut and soybean treatments were 24% and 44% lower (*P* < 0.05), respectively, compared to the bare fallow. This result indicates that legume cropping supressed the proliferation of known ammonia oxidisers, which corroborated the higher concentrations of NH_4_^+^ (*r* = −0.75, *P* < 0.001; *n* = 12) and lower concentration of NO_3_^−^ (*r* = 0.67, *P* < 0.05; *n* = 12) in both legume cropping treatments compared to bare fallow (Table 1). Recently, a study examining legume cropping effects on soil N cycling pathways also showed that the abundance of ammonia oxidisers decreased in the rhizosphere during maize-faba bean intercropping^25^.

**Table 3:** Relative abundance of ammonia oxidisers and nitrite oxidisers in bare fallow, peanut cropping and soybean cropping treatments. Data represent averages ± SE of four replicates.

Genus identification (Phylum)	Bare	Peanut	Soybean
	Relative abundance (%)		
**Ammonia oxidising archaea**			
*Nitrosopumilus* (Thaumarchaeota)	0.03 ± 0.02^a^	0.00 ± 0.00^a^	0.00 ± 0.00^a^
*Nitrososphaera* (Thaumarchaeota)	0.48 ± 0.11^a^	0.24 ± 0.07^b^	0.14 ± 0.07^b^
**Ammonia oxidising bacteria**			
*Nitrosomonadaceae* (Proteobacteria)	0.03 ± 0.02^a^	0.02 ± 0.01^a^	0.06 ± 0.03^a^
**Sum**	**0.54** ± **0.15** ^a^	**0.26** ± **0.08** ^b^	**0.20** ± **0.10** ^b^
**Nitrite oxidising bacteria**			
*Nitrospira* (Nitrospirae)	0.28 ± 0.07^a^	0.29 ± 0.06^a^	0.25 ± 0.06^a^
*Nitrobacter* (Proteobacteria)	0.04 ± 0.02^a^	0.01 ± 0.01^a^	0.00 ± 0.00^a^
**Sum**	**0.32** ± **0.09** ^a^	**0.30** ± **0.07** ^a^	**0.25** ± **0.06** ^a^

All taxa listed are genus level except for the family *Nitrosomonadaceae*. Numbers within a row followed by different letters are significantly different (ANOVA, LSD post hoc test, at *P* < 0.05).

### Abundance of *amoA* Gene

**Figure 2:**
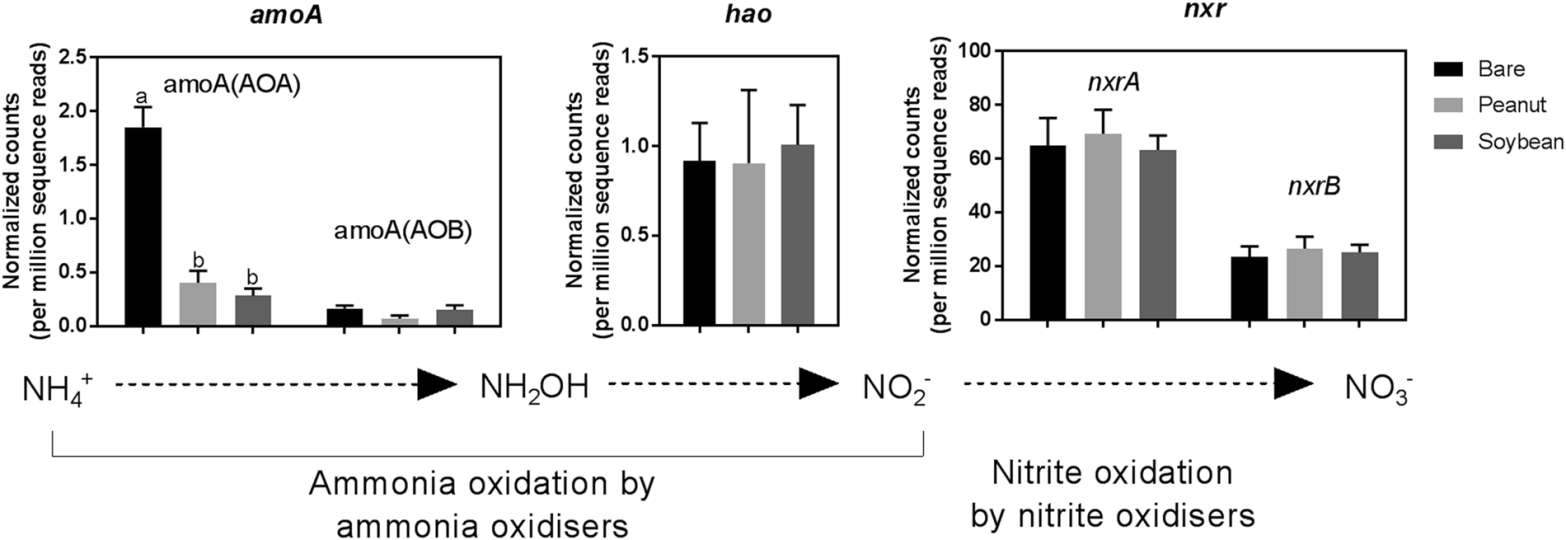
Effects of legume crop rotation on the abundance of nitrification genes. Read counts were normalised by sample-specific number of sequence reads to compare between samples. The genes and their encoded enzymes are: *amoA*(AOA), archaeal ammonia monooxygenase; *amoA*(AOB), bacterial ammonia monooxygenase; *hao*, hydroxylamine oxidoreductase; *nxrA*, nitrite oxidoreductase α subunit; *nxrB*, nitrite oxidoreductase β subunit. Error bars denote standard error of the mean. Different letters in each panel indicate significant differences at *P* < 0.05 (ANOVA, LSD post hoc test).

To assess abundances of the *amoA* gene, which encodes the active site of ammonia monooxygenase enzyme that oxidises ammonia to hydroxylamine in the first step of nitrification^26^, shotgun sequencing data were first assigned KOs with reference to the Uniref100 database. A total of 59,662,311 sequences (12.5% of the 475,846,598 reads) were classified into 14,391 KOs, in which there were significantly more archaeal than bacterial *amoA* sequences (*P* < 0.001; Fig. 2). In addition, the relative abundances of archaeal *amoA* gene in the peanut and soybean soils were only about 22% and 15%, respectively, of that in the bare fallow soil (*P* < 0.05; Fig. 2). As the total microbial dsDNA in the peanut (4.9 ± 0.5) and soybean (6.2 ± 0.2) cropping soils was 1.6 and 2.0 times higher, respectively, than in bare fallow (3.1 ± 0.4), the total abundances of archaeal *amoA* in the peanut and soybean treatments were 35% and 30% of that in the bare fallow, respectively (*P* < 0.05). These *amoA* abundance profiles corroborated (*r* = 0.77, *P* < 0.01; *n* = 12) the 16S-based measurements of AOA and AOB (Table 3). Predominance of archaeal over bacterial *amoA* genes has also been observed in other agricultural soils, particularly acidic soils^25,27–29^. However, the lower abundances of AOA and archaeal *amoA* gene in the root zone of the legume cropping soils compared to bare fallow differed from the findings in a paddy rice field where AOA was more abundant in the rhizosphere than in bulk soil^28^.

### Abundance of *hao* Gene

The abundances of hydroxylamine oxidoreductase (*hao*), which oxidises hydroxylamine to nitrite, were similar in the legume cropping and the bare fallow soils (Fig. 2). Unlike *amoA*, *hao* is unique to autotrophic ammonia oxidising bacteria (AOB) and is lacking in ammonia-oxidizing archaea^30^. Consistent with this result, relative abundances of the autotrophic AOB *Nitrosomonadaceae* was similar in the three treatments (Table 3).

## Conclusions

Our results demonstrated that soybean or peanut rotation between sugarcane crop cycles can alter soil microbial community composition, increase bacterial and archaeal biomass but reduce the abundances of ammonia oxidisers and *amoA* genes encoding ammonia monooxygenase. These results invite further studies on (i) mechanisms for the inhibitory effects of crop rotation on the nitrifying community including changes in soil biochemical and biophysical conditions due to crop root activities and exudates; (ii) temporal variation in the microbial composition and gene abundance during the fallow period and the subsequent sugarcane cropping season; and (iii) relationships between changes in the nitrifying microbial communities and nitrification rates under field conditions.

## Materials and Methods

### Field Experiment and Soil Collection

The field experiment was established at Bundaberg, Queensland, Australia (S 25°01′31.8″ E 152°22′47.6″) during the fallow period (October 2015 to July 2016) between two sugarcane crop cycles. This site had been grown with sugarcane crop for more than ten years. The previous sugarcane crop was fertilised with about 150 kg N/ha as urea in October 2014 and was harvested in October 2015 with cane trash (plant residues) retained on the ground. The soil is a loamy sand containing 10% clay (<2 µm), 12% silt (2–20 µm) and 78% sand (>20 µm), 10.5 mg organic carbon g^−1^ and 0.8 mg total N g^−1^ in the 0–20 cm depth.

The long-term (1959–2017) annual mean temperature in this subtropical region is 21.6°C (Bundaberg Aero Station, the Bureau of Meteorology, Australia), with the lowest monthly mean temperature in July (16.2°C) and the highest in January (25.9°C). Mean annual rainfall is 1027 mm, with ca. 56% of rainfall received from December to March. During the 132-day period between legume crop planting to soil sampling in this study (17 December 2015 to 27 April 2016; see below), 549 mm of rainfall (502 mm in the first one and half months) and 175 mm of spray irrigation (7 events × 25 mm in the last two months) were received.

Limestone powder was applied at 2.0 t ha^−1^ on 27 November 2015 to correct low soil pH (5.3 in 1:5 soil and water suspension) and high aluminium saturation (15% of CEC). A fertiliser blend was surface-applied at 12 kg N ha^−1^, 26 kg P ha^−1^, 57 kg K ha^−1^, 15.6 kg S ha^−1^ and 19 kg Ca ha^−1^ and then incorporated into soil with a rotary hoe. There were three management treatments: bare fallow (control), peanut (*Arachis hypogaea*) cropping and soybean (*Glycine max*) cropping, arranged in a randomised block design with four replicates per treatment. Shortly after the fertiliser application, the legume crops were planted in dual rows 90 cm apart on raised beds (~120 cm wide) on 17–18 December 2015, with peanut or soybean inoculants applied into the planting furrows to ensure adequate nodulation.

Soil samples were collected from the 0–10 cm depth in the crop root zone or similar positions in the bare fallow on 27 April 2016, approximately at the maximum biomass stage of the legume crops. The rationale for sampling at this time is that there should be a best chance to detect possible effects of legume cropping on the soil microbial community in the rhizosphere at this stage^31^. Eight separate samples of soil were taken from each plot and pooled (~300 g), resulting in four replicates per treatment. The soil samples were transported to the laboratory on the same day in insulated boxes filled with ice blocks, stored in a fridge at 4°C overnight and sieved through a sterilised 2 mm sieve. Sub-samples were air-dried for physical and chemical analyses or stored at −20°C for DNA isolation.

### Analyses of Soil Physico-Chemical Properties

Soil moisture content was determined by oven-drying ~50 g of the moist samples for >24 h at 105°C and recording weight loss. NH_4_^+^-N and NO_3_^−^N contents were determined using the 2 M KCl extraction and colorimetric spectrometry method^32^. Soil pH was measured in 1:5 soil:water extracts with calibrated electrodes at about 25°C. Total organic C and N contents in soil were determined by the Dumas combustion method using a TruMac^®^ CN analyser (LECO, St Joseph, MI, USA). Primary particle size distribution was determined using the pipette method^33^.

### DNA Extraction and Shotgun Metagenome Sequencing

We used shotgun metagenomic sequencing to determine the relative abundances of nitrifying microorganisms and nitrification-related genes in the soil samples. Total dsDNA was extracted from 0.25 g of soil using the PowerSoil^®^ DNA isolation kit following manufacturer’s instructions (Mo Bio Laboratories, Inc., Carlsbad, CA, USA). DNA libraries were prepared using an Illumina® Nextera XT Library Prep Kit following manufacturer’s protocol. The DNA libraries were paired-end sequenced on an Illumina NextSeq500 sequencer, producing 150 bp read lengths.

### Metagenome Analysis

All primary sequencing data were deposited in GenBank under accession number SRP075781. Read quality was assessed using FASTQC v0.10.1 (http://www.bioinformatics.babraham.ac.uk/projects/fastqc/). Forward reads from each sample were aligned against reference protein sequences in the UniRef100 database (2015_10 release) using DIAMOND V0.7.9^34^. KEGG Orthology (KO) was then assigned according to the best alignment matches and a KO-by-sample count table was created. These KO counts were then normalised to counts per million sequence reads for each sample to account for sequencing depth.

### Microbial Community Profile Data Processing

Community composition was determined by searching for 16S rRNA gene sequences in metagenomic sequence data using a 16S rRNA gene Hidden Markov Model. Putative 16S rRNA sequences were then assigned taxonomy by phylogenetic placement in a reference 16S rRNA gene tree (Greengenes May 2013 release)^35^ using pplacer v2.6.32^36^. The 16S rRNA gene sequence search and phylogenetic placement procedures were performed as implemented in GraftM v0.9.5 (https://github.com/geronimp/graftM). A site-by-species operational taxonomic unit (OTU) count table was constructed from the GraftM output and counts were converted to relative abundances with adjustments for lineage-specific 16S gene copy number variation using CopyRighter V0.46^37^. Variation in community composition under different fallow treatments was assessed using permutational multivariate analysis of variance (PERMANOVA), and visualised using an ordination of relative abundance data (Redundancy analysis, RDA).

### Classifying *amoA* sequences

Sequences assigned to the KO K10944 (*amoA*/*pmoA*) were placed in a phylogenetic tree containing reference *pmoA* and *amoA* gene sequences from various bacterial and archaeal taxa using pplacer V2.6.32. Their putative taxonomies and read counts were collated into a counts table, which was then normalised for sequencing depth. The phylogenetic placement procedures were performed as implemented in GraftM V0.10.1 (https://github.com/geronimp/graftM).

### Quantitative Polymerase Chain Reaction (qPCR)

qPCR analysis was performed to quantify absolute bacterial and archaeal abundances using the 16S 1406 F/1525 R primer set (0.4 µM): F-GYACWCACCGCCCGT and R-AAGGAGGTGWTCCARCC. The PCR was set up using 5 µl of 2X SYBR Green/AmpliTaq Gold DNA Polymerase mix (Life Technologies, Applied Biosystems), 4 µl of microbial template DNA and 1 µl of primer mix. The rpsL F/R primer set (0.2 µM), used for inhibition control, amplifies *Escherichia coli* DH10B only: F-GTAAAGTATGCCGTGTTCGT and R-AGCCTGCTTACGGTCTTTA. Three dilutions of 1/50, 1/250 and 1/500 (microbial template DNA, 16S 1406 F/1525 R primer set) as well as an inhibition control (*E. coli* DH10B genomic DNA, rpsL primer set) were run in triplicate for each sample. The PCR was run on the ViiA7 platform (Applied Biosystems) including a cycle of 10 min at 95°C (AmpliTaq activation) and 40 cycles of 15 s at 95°C followed by 20 s at 55°C and 30 s at 72°C. A melt curve was produced by running a cycle of 2 min at 95°C and a last cycle of 15 s at 60°C. The cycle threshold (Ct) values were recorded and analysed using ViiA7 V1.2.1 software.

### Statistical Analysis

All statistical analyses of bioinformatics were implemented using R V3.2.2^38^ with the vegan package^39^. Community composition was visualised using Redundancy analysis (RDA) with soil moisture, electrical conductivity, pH, and ammonia and nitrate concentrations fitted onto the RDA ordination as vectors. Bacterial and archaeal species richness and evenness were calculated using QIIME V1.8.0^40^ and represented using the Chao1 metric and Shannon’s index, respectively. To assess the differences among treatments, statistical analyses were performed using ANOVA, LSD post hoc test (GraphPad Prism4, GraphPad Software, Inc., San Diego CA, USA).
